# Supplementary material for: Conspicuous corruption: Evidence at a country level
Source: PLoS One. 2021 Sep 1;16(9):e0255970. doi: 10.1371/journal.pone.0255970 (PMC8409654; doi:10.1371/journal.pone.0255970)
Supplement: Attachment — Submitted filename: plos_rebuttal_letter.pdf [file pone.0255970.s001.pdf]

Response to the reviewers

We would like to take the opportunity to thank the reviewers for their critical assessment of our work. In the following we address their concerns point by point.

Reviewer 1

Reviewer Point P 1.1 — The main idea and conclusion is done perfectly and where is the analyze was convenient, keep it up and looking forward for more upgrades and more technical updates for the same topic and it's a big source for more research about it.

Reply: Thank you.

Reviewer 2

Review of "Conspicuous Corruption"

Thank you for this really interesting article about "conspicuous corruption" in Greece. I found the data and approach to be novel, and it was an interesting and well-written article. I believe the article should be published as it will be an important contribution to the literature on corruption. However, there are a few areas the authors should consider addressing in order to improve the article and strengthen the claims being made:

Reviewer Point P 2.1 — The authors should make clear straight from the beginning that what they can offer is evidence of an association between corruption and vanity plates, but no clear causal evidence using this data source about so-called "vanity plates" being the result of corrupt behavior. The authors do state this in the end, but it really should also appear up front.

Reply: We have moved (and slightly reworded) the relevant statement to the start of the paper, at the end of the Introduction section.

Reviewer Point P 2.2 — The theory is quite thin, and discusses Veblen goods but not the literature on corruption drivers. I'd rather like to see the discussion about corruption that appears on page 6, and in particular the discussion about the author's concept of "conspicuous corruption", moved up to appear prior to the methodology and data section.

Reply: We moved our definition of "conspicuous corruption" in the Introduction section. We kept the bulk of the discussion and the literature at the end, feeling that this suits better the PLOS style which places all dicsussion at the end, as detailed in `https://journals.plos.org/plosone/s/submission-guidelines`. Moreover, we enriched the discussion through two relevant references [1, 2].

Reviewer Point P 2.3 — I think the authors need to do a bit more to convince the reader that vanity plates are truly Veblen goods. First, if I understand it correctly, since there are no legal means to obtain vanity plates in Greece, this means that the trade in vanity plates is a black

market and vanity plates are illegal goods, not "normal" luxury items. Is it the luxury car or the vanity plate that is the Veblen good, or is it both? Is a vanity plate a Veblen good if it does not also appear on a luxury car? Second, a vanity license plate seems like a rather cheap Veblen good, and without more knowledge about how the Greek population perceives vanity plates it is hard to understand the true value of vanity plates in Greek society. We would really need to know more about the pricing of vanity plates to truly understand them as Veblen goods: what is the going rate for such a plate?

Reply: We show that it is highly unlikely that they are obtained legally; however, after their acquisition, they are not illegal. A car owner cannot be arrested for driving a car with a vanity plate, unless there is evidence that it was obtained illicitly. The first question raised under this point is whether a vanity plate *by itself* would count as a Veblen good; i.e., would it be considered a luxury item if it was displayed on a non-luxury car? Of course a vanity plate will not turn a middle-of-the-road model to a luxury vehicle. However, it might have a small effect: researchers found that in The Netherlands, a new license plate format, with absolutely no intrinsic value, increased a car's price by about 4% [3]. We have modified the beginning of the Discussion section accordingly, to allow for more nuance in our definitions. Regarding the second question raised under the point, the going rate is a few hundred of Euros, as we state in the beginning of the Discussion section. We have also made a few additional points on the value of the illicitly acquired vanity plates in the (new) Conclusions section.

Reviewer Point P 2.4 — The last question I ask under P 2.3 points to a real gap in this article, but one which may be too large to actually address in the article (in which case, perhaps the authors could point to this as a research gap, and/or do more work themselves in future work): this is more understanding of the Greek context and what is considered to be luxury in the context. At a very basic level, it would be useful to know how the licensing process works. Could it be, for instance, that there are legal loopholes that allow, for instance, purchasers of new cars to request any license they want, and that is why we see the association between car type and vanity plates? On a related point: while I like the concept of "conspicuous corruption", doesn't this only work if everyone knows that vanity plates are widely understood to be an ill-gotten good? Is it in fact the case that Greek people generally understand vanity plates to be acquired through corrupt means? Also—the authors refer to a Wikipedia list to ascertain what is a luxury vehicle, but isn't such a list dependent on context? For instance, the author argue that a Smart car would be considered a luxury vehicle in Greece, but this would not necessarily be the case in other places where Smart Cars are more common and even looked down upon. Another issue is understanding the value of vanity plates—does the Greek population generally consider vanity plates to be a luxury good? Third, more qualitative evidence about how the black market in vanity plates in Greece works would be useful to bolster the author's claims that they are, in fact, considered luxury goods. On page 6, the authors talk about "rumors" regarding the price of vanity plates, but surely they could also conduct some interviews or find newspaper articles to support this claim?

Reply: We added some explanation of how the racket works in the beginning of the Discussion section. We have no proof that the Greek people in general understand that vanity plates are acquired through corrupt means (although we strongly suspect so). This points at the possibility that the value associated with a vanity plate is not only based on its scarcity, but is also works as a social marker that the owner "knows the ropes", or even is "above the rules". Smart cars are not considered luxury cars in Greece, but they are a status symbol for the design-conscious. We have clarified that, and it certainly is context-dependent. We have added the observation in the Discussion section. Regarding the last point, we added

a short description of how the system works, giving a reference to a newsparer article (in Greek) [4] in the beginning of the Discussion section. We also describe briefly how the racket apparently works in the context of the licensing system.

Reviewer Point P 2.5 — Data: what is the timeframe of the data? Details are provided as to when the data was acquired and the number of observations, but not the range of years and geographical locations covered.

Reply: The dataset covers all registered cars in Greece up to 2017-03-31; we added the clarification in the dataset description.

Reviewer Point P 2.6 — It would be useful to provide some real-world examples of cars when discussing engine size. What kind of luxury car would be found in the larger vs. smaller engine size categories discussed on page 4? How do we know these larger engine sizes are not trucks, i.e. working vehicles?

Reply: The record for each car contains an attribute corresponding to the vehicle type and vehicle use. We have kept only vehicles that are registered as "private" (working vehicles are usually registered as "public" in Greece) and whose type is "passenger vehicle" or "mixed use". We have added the clarification in the dataset description in the text.

Reviewer 3

First, I would like to thank you for the opportunity to review this paper. In general. I believe the authors have investigated a good research topic, entitled, "Conspicuous Corruption: Evidence at a Country Level." The research area lies in my area of interest. I have published articles related to this topic in leading journals. I have enjoyed reading and evaluating this article as it matches my research interests.

This article describes its objective to explore, analyze and evaluate ideas and perceptions of "Conspicuous Corruption". The study describes that general public with wealth can show their status by consuming a particular product or experience Purchase; this is known as "apparent consumption": this practice is widespread and explained the market characteristics of the entire category of goods, Veblen goods, demand increases with its price. Commodities' value lies in their distribution among populations—the rarer they are, the more desirable they become. At the same time, higher incomes tend to be associated with higher status levels, which has been studied in its relationship to unethical behavior. Here, we introduce research Show how status symbols, immoral behavior, and wealth come together displays illegal as a status symbol. We collect evidence at a large national-level the scale of a particular form of consumption of unlawful benefits for status purposes.

The study has focused on a critical issue of society. However, I have some suggestions for the authors to enhance this work quality. I am recommending your research for publication. It is a good topic; however, you need to work on my suggestions to reach scientific merit. Make changes one by one as suggested.

Reviewer Point P 3.1 — Title: Revise the title and cover it with a theme reflecting the central idea of the study. Your title does not display the real sense of the main study.

Reply: We respectfully disagree with the change in title; we believe that the title should reflect the main finding of our study, which indeed is the phenomenon we term "conspicuous corruption" and for which we do gather evidence at a whole country level.

Reviewer Point P 3.2 — Introduction section: The introduction is not strong. Expand your introduction to about 800 words. Discuss research gaps identified from the literature. In my opinion, here two points could very interesting in this study. (1) How can social media play a leading role in educating people about this bad habit? (2) How corporate social responsibility (CSR) can make people sensible to perform their good social duties and contribute to society.

I strongly suggest you build your study with the idea. Your research will become excellent as you have already explored a great idea. The whole world is facing such issues in society; however, it is becoming common in some developing countries. These rich people can spend this wealth for the social good to become immortal. I am suggesting outstanding studies published in leading journals. Please read these studies, improve your introduction, and cite these articles to enhance your work quality.

Su, Z., McDonnell, D., Wen, J., Kozak, M., Abbas, J., Šegalo, S., Li, X., Ahmad, J., Cheshmehzangi, A., Cai, Y., Yang, L., & Xiang, Y.-T. (2021, 2021/01/05). Mental health consequences of COVID-19 media coverage: the need for effective crisis communication practices. Globalization and Health, 17(1), 4. `https://doi.org/10.1186/s12992-020-00654-4`

Abbas, J., Aman, J., Nurunnabi, M., & Bano, S. (2019). The Impact of Social Media Lon earning Behavior for Sustainable Education: Evidence of Students from Selected Universities in Pakistan. Sustainability, 11(6), 1683. `https://www.mdpi.com/2071-1050/11/6/1683`

Hussain, T., Abbas, J., Wei, Z., Ahmad, S., Xuehao, B., & Gaoli, Z. (2021). Impact of Urban Village Disamenity on Neighboring Residential Properties: Empirical Evidence from Nanjing through Hedonic Pricing Model Appraisal. Journal of Urban Planning and Development, 147(1), 04020055. `doi:10.1061/(asce)up.1943-5444.0000645`

Abbas, J., Mahmood, S., Ali, H., Ali Raza, M., Ali, G., Aman, J., . . . Nurunnabi, M. (2019). The Effects of Corporate Social Responsibility Practices and Environmental Factors through a Moderating Role of Social Media Marketing on Sustainable Performance of Business Firms. Sustainability, 11(12), 3434.

Reply: We have included the related literature in the Discussion section. Please note that this follows the guidelines to PLOS authors regarding the manuscript organization at `https://journals.plos.org/plosone/s/submission-guidelines`.

Regarding the suggested work, our work aims at exposing a phenomenon that predates social media, so the references to media coverage is not relevant. It also predates COVID-19, so that reference is not relevant. Moreover, it has no relation to urban planning, or indeed to any geography-related issues, as the dataset comprises vehicle registration throughout Greece.

Reviewer Point P 3.3 — Literature section: The literature section needs improvement. I suggest the authors to look into the suggested studies to improve literature section. Build your idea how innovative strategies can bring change in the government organizations to change public. The authors add the latest citations to the literature and method sections to enhance the study's quality. Cite these studies in the literature to enhance the quality of your work.

Abbas, J. (2021). Crisis management, transnational healthcare challenges and opportunities: The intersection of COVID-19 pandemic and global mental health. Research in Globalization, 100037. https://doi.org/10.1016/j.resglo.2021.100037

Local Burden of Disease, H. I. V. C. (2021, 2021/01/08). Mapping subnational HIV mortality in six Latin American countries with incomplete vital registration systems. BMC Medicine, 19(1), 4. https://doi.org/10.1186/s12916-020-01876-4

Abbas, J., Zhang, Q., Hussain, I., Akram, S., Afaq, A., & Shad, M. A. (2020). Sustainable Innovation in Small Medium Enterprises: The Impact of Knowledge Management on Organizational Innovation through a Mediation Analysis by Using SEM Approach. Sustainability, 12(6). https://doi.org/10.3390/su12062407

Reply: We struggle to see the relevance of the suggested publications with our work. In particular our work is not related to:

- COVID-19 and global mental health,
- HIV mortality, nor
- sustainable innovation.

Regarding the suggestion "how innovative strategies can bring change in the government organizations to change public", the goal of our research is not strategy, but the identification and exploration of a particular form of corruption that impacts both the public and the private sector.

Reviewer Point P 3.4 — Methods and results: I suggest adding demographic table by covering education level, age, income level and regions. See the suggested study and explore if religiosity level can motivate people to contribute to the society. You can add graphical presentation of your findings. See these studies to improve your work and cite them in the methods and results sections.

Abbasi, K. R., Abbas, J., & Tufail, M. (2021, 2021/02/01/). Revisiting electricity consumption, price, and real GDP: A modified sectoral level analysis from Pakistan. Energy Policy, 149, 112087. https://doi.org/10.1016/j.enpol.2020.112087

Aman, J., Abbas, J., Nurunnabi, M., & Bano, S. (2019). The Relationship of Religiosity and Marital Satisfaction: The Role of Religious Commitment and Practices on Marital Satisfaction Among Pakistani Respondents. Behavioral Sciences, 9(3), 30. https://www.mdpi.com/2076-328X/9/3/30

Abbasi, K. R., Hussain, K., Abbas, J., Adedoyin, F. F., Shaikh, P. A., Yousaf, H., & Muhammad, F. (2021). Analyzing the role of industrial sector's electricity consumption, prices, and GDP: A modified empirical evidence from Pakistan [J]. AIMS Energy, 9(1), 29-49. https://doi.org/10.3934/energy.2021003

Reply: The dataset does not contain demographics. There is no relation to religiosity levels (and no way to determine them), nor to electricity consumption or marital satisfaction. We already include graphs, plots, and photos in the manuscript.

Reviewer Point P 3.5 — Discussion section: Make a separate heading for discussion section, build it on 1000 words, and improve this section. It is short in your study. It should be around one page and a half. Make it strong. See the recommended studies and improve your sections.

Reply: Concerning the recommended studies, please see the answers above; we struggled to see their relevance to our work. Regarding the heading and the format, we followed the guidelines to the authors for the PLOS manuscript organisation as set out in `https://journals.plos.org/plosone/s/submission-guidelines`.

Reviewer Point P 3.6 — Conclusion: Make a separate heading for conclusion and don't mix it with the discussion section. Your conclusion should be based on minimum 500 words.

Reviewer Point P 3.7 — We added such a section in the end, also accommodating some of the points / clarifications raised by the other reviewers.

Reviewer Point P 3.8 — Implications: Make a separate heading for implications of your study. I suggest adding implications heading and briefly explain it in this section.

Reply: As the PLOS guidelines do not specify a separate Implications sections, we refrained from doing so.

Reviewer Point P 3.9 — Limitations: Make a proper heading and discuss it adequately. Highlight the study's scientific contribution to scientific knowledge. The authors should explain how this study offers useful insights to the researchers of the tourism industry in the discussion section. The English level needs corrections to meet scientific merit for publication. I accept and endorse this manuscript for publication after minor modifications, as suggested.

Reply: We discuss the limitation of whether this is an isolated phenomenon in a particular country at length in the Discussion section. The PLOS guidelines do not specify a separate Limitations sections. The paper has no relationship with the tourism industry. We have proofread the text carefully and corrected a few remaining issues; if any more infelicities do remain, we would be grateful if they could be pointed out.

Reviewer 4

The manuscript addresses a significant issue. The presentations of ideas are demonstrative. However, some comments need to be taken into account:

Reviewer Point P 4.1 — The main point of the manuscript is the relationships between corruption and wealth in Greece. This was apparent from the title. The authors affirmed that when asserted that the study shows "status symbols, unethical behavior, and wealth, combine to produce the display of illegality" (p1). However, the data is confined to one good "car plates". I do not think it sufficient to have this data as a generalization basis. The manuscript should have gathered and analyzed data of more than one type of accessories to conclude the relationship between illegal behavior and wealth. The other option is to minimize the scope of research (through title and discussion) to have compatibility between title, scope, and data analysis.

Reply: We amended the abstract to reflect more accurately the scope of our research. We also made clearer that we are not referring to illicit goods, but to goods that are illicitly acquired—a vanity plate, no matter how it has been acquired, it is not illegal per se. It is a valid license plate. In the introduction

we make it clear that we are talking about one particular kind of goods: "We examine whether vehicle owners engage in rule-breaking in order to acquire a particular form of a positional good: a vanity plate for their vehicle."

Reviewer Point P 4.2 — Corruption, illegality, and unethicality have been used interchangeably throughout the manuscript. Every term has its meaning which may defer from others in the light of the manuscript hypothesis. In this regard, the manuscript uses some references related to unethical behavior with wealth as a support to his propositions about law-breaking activities. I suggest that authors should review the use for more coherence.

Reply: Following your suggestion, we have gone through the manuscript carefully:

- We now use the term "unethical" only relating to results of work in the literature.
- We use the term "corruption" only as in "conspicuous corruption", except from our discussion of corruption in general in the Discussion section (where we start that part of the discussion by "In terms of corruption in general", so the distinction should be clear).

Reviewer Point P 4.3 — The manuscript should have elaborate more in the materials section about the regulations of the acquirement of plate numbers. For instance, the legality of ownership transfer of plates, which is available in many countries worldwide, may affect the whole hypothesis of the manuscript.

Reply: These are good points. License numbers are not transferable. We have added details on the acquisition process in the Materials section and details on how the process can be subverted in the Discussion section.

Reviewer Point P 4.4 — On page 2, the manuscript states that "we examine whether vehicle owners engage in rule-breaking in order to acquire a particular form of a positional good". I did not this examination. Moreover, the manuscript states on page 6 "Conspicuous corruption works because it cannot be proved that the law has been broken".

Reply: It is correct that "engage in rule-breaking" does not reflect accurately what is happening; after all, as we now explain in the Materials and the Discussion sections, license numbers are often acquired by car dealerships, so it is not the owners themselves who carry out the rule-breaking, but the sellers, as a (paid) service to their customers. However, that does not absolve the owners, as they do have to pay for the vanity plate. So it is more accurate to change the sentence to "We examine whether vehicle owners are involved in rule-breaking in order to acquire a particular form of a positional good: a vanity plate for their vehicle." Concerning the proof that the law has been broken, we have rephrased it to "Conspicuous corruption works because it cannot be proved that the law has been broken in a particular instance after the fact; a license plate is by itself legal, even though it may have been illegaly acquired."

Reviewer Point P 4.5 — On page 5, the study cites Wikipedia for a piece of information. I do not think this is an authoritative reference.

Reply: Our use of Wikipedia does not involve taking an article as an authoritative reference for a particular subject, but using its category of "Luxury vehicles" to sieve the luxury cars; see `https:`

//en.wikipedia.org/wiki/Wikipedia:Categorization. In order to make sure that the category is not off the mark, we cross-checked with the Euro Car Segments, which are in use in Europe for categorizing vehicles. In particular, we examined the "F: luxury cars" segment, by considering the sales in these segments for 2020, as reported by the Hellenic Association of Motor Vehicle Importers-Representatives. We found that all the F-segment cars sold in Greece in 2020 belonged indeed into the Wikipedia "Luxury vehicles" category, apart from Tesla and Ford Explorer cars. As Ford Explorer is not a brand (it is a brand and a model); therefore we only added Tesla in the examined luxury vehicles. This did not alter the statistical results.

Reviewer Point P 4.6 — On pages 5–6, the authors state that "After some investigation, it appears that the market for vanity plates in Greece is an open secret, the cost for obtaining a desirable number running to a few hundred Euros." These facts should be proved by statistics or citations.

Reply: An internal audit conducted by the competent ministry has uncovered several irregularities that demonstrate the *modus oprandi* for handing out vanity plates. We have asked for the audit report through a freedom of information request, which was however rejected, due to the report's confidentiality. However, the report has leaked, and we were thus able to add a reference to it from a newspaper article (in Greek, our translation of the most relevant part) in the Discussion section.

References

[1] Kleven HJ, Knudsen MB, Kreiner CT, Pedersen S, Saez E. Unwilling or Unable to Cheat? Evidence From a Tax Audit Experiment in Denmark. Econometrica. 2011;79(3):651–692.

[2] Alstadsæter A, Johannesen N, Zucman G. Tax Evasion and Inequality. American Economic Review. 2019;109(6):2073–2103.

[3] Kooreman P, Haan MA. Price Anomalies in the Used Car Market. De Economist. 2006;154(1):41–62.

[4] Mandravelis P. The Privatisation of License Numbers; 17/01/2014. The Kathimerini Newspaper (in Greek).
