# Supplementary material for: Conspicuous corruption: Evidence at a country level
Source: PLoS One. 2021 Sep 1;16(9):e0255970. doi: 10.1371/journal.pone.0255970 (PMC8409654; doi:10.1371/journal.pone.0255970)
Supplement: Attachment — Submitted filename: plos_rebuttal_letter_2.pdf [file pone.0255970.s002.pdf]

Response to the reviewers

We would like to take the opportunity again to thank the reviewers for their critical assessment of our work. There were two remaining points by Reviewer #4. We addressed them in the manuscript and we give details below.

Reviewer 1

The manuscript addresses a significant issue. The presentations of ideas are demonstrative. However, some comments need to be taken into account:

Reviewer Point P 1.1 — Regarding comment No 2. I mentioned that "Corruption, illegality, and unethicality have been used interchangeably throughout the manuscript". The authors responded by stating:

> Following your suggestion, we have gone through the manuscript carefully:
>
> - We now use the term "unethical" only relating to results of work in the literature.
> - We use the term "corruption" only as in "conspicuous corruption", except our discussion of corruption in general in the Discussion section (where we start that part of the discussion by "In terms of corruption in general", so the distinction should be clear).

I noticed that there is still an overlap in P2 "researchers have shown a link between unethical or illegal behaviour and higher wealth".

Reply: We used "unethical or illegal behaviour" to cover all three of the references mentioned in the sentence. We have now changed the phrase. The term "unethical" refers to the first two references [1, 2], as also shown by their titles. The term "illegal" refers to the third reference, which concerns yielding right of way to pedestrians; the authors point out that "Drivers simply may not know that they are legally required to yield to pedestrians." [3].

Reviewer Point P 1.2 — Concerning my comments No 5. therein I stated, "On page 5, the study cites Wikipedia for a piece of information. I do not think this is an authoritative reference". I am afraid that the authors' justification is not convincing. Multiple scientific works conceptualised "luxury cars". For instance : MAHMOUDIAN, ALI. "Role of global suppliers in luxury car industry." (2019).

Reply: We have gone through the bibliography carefully. There does not seem to exist a single widely accepted definition of what cars fall into the luxury category, apart from focusing on certain well-known brands:

- "As conspicuous luxury car, a Porsche Boxster was selected, while a Ford Fiesta was chosen as inconspicuous nonluxury car." [4]
- "In this vein, three brands (BMW, Audi and Mercedes-Benz) are chosen to be considered in this study due to the fact that they are the most representative to the E (executive cars), F (luxury cars) and S (sport coupés) car segments according to [5]" [6].

- In [7] the researchers use the following luxury car brands: Mercedes-Benz, Bentley, Jaguar, Lexus, Ferrari, Porsche, Cadillac, BMW, Lamborghini, Rolls-Royce.

- "The notion of a luxury car varies widely in the literature. For example, Rosecky and King [8] cite five different definitions and limit their study to owners of Mercedes, BMW, Jaguar, Cadillac, Lincoln, Lexus, Infinity, and Acura brands. Is a $50,000 Ford a luxury? What about a $20,000 BMW?" citestrach:2006.

- In [9] "status-oriented cars" include Mercedes-Benz, BMW, and Audi; "exclusive cars" include Aston Martin, Bentley, Bugatti, Ferrari, Jaguar, Lamborghini, Lotus, Maserati, Maybach, Porsche, and Rolls Royce.

- In a study of "Conspicuous consumption and satisfaction" the luxury cars were Ferraris and Porsches [10].

Note that we found MAHMOUDIAN, ALI "Role of global suppliers in luxury car industry." (2019) published as an MSc thesis. In that thesis "we use the term High Luxury car Segment (HLS) as coined by Aston-Martin in their annual report (2018).Though it is not a term by academia, it is a differentiating term by industry's experts (i.e. Aston Martin)". Reading that annual report we find that the segment includes Aston Martin, Bentley, Ferrari, Lamborghini, McLaren, and Rolls-Royce. The thesis itself uses Aston Martin, Ferrari, Maserati, Porsche, Lamborghini, Bentley, Rolls Royce, and Bugatti.

Returning to our own research, if we use the F-G segment cars (the Greek sales data report "F-G" segment instead of plain "F" segment), our dataset includes the following brands for the years 2000-2020:

- Audi
- BMW
- Bentley
- Cadillac
- Chrysler
- Citroen
- Ford
- GM
- Hyundai
- Jaguar
- Lancia
- Land Rover
- Lexus
- Maserati

- Mercedes
- Nissan
- Opel
- Peugeot
- Porsche
- Renault
- Rolls-royce
- Saab
- Tesla
- Volkswagen
- Volvo

From the above, the following cars do not appear as luxury in the Wikipedia category:

- Chrysler
- Citroen
- Ford
- GM
- Hyundai
- Nissan
- Opel
- Peugeot
- Renault
- Saab
- Tesla
- Volkswagen

Except for Tesla and Saab, these are indeed brands that produce luxury models, but are not mainly or exclusively luxury brands, so we removed them. Also, note that the F-G segment cars do not include luxury brands such as Ferrari, which are categorized as sports cars in the sales reports.

We carried out our analysis using the F-G segment data and we found, as before, that the probability of a vanity plate appearing on a luxury car is about double that of the probability of a vanity plate on all cars. We now report this result in the paper and also mention that the situation is the same if we use the Wikipedia categories.

References

[1] Gino F, Pierce L. The Abundance Effect: Unethical Behavior in the Presence of Wealth. Organizational Behavior and Human Decision Processes. 2009;109(2):142–155. doi:10.1016/j.obhdp.2009.03.003.

[2] Piff PK, Stancato DM, Côté S, Mendoza-Denton R, Keltner D. Higher social class predicts increased unethical behavior. Proceedings of the National Academy of Sciences. 2012;109(11):4086–4091. doi:10.1073/pnas.1118373109.

[3] Coughenour C, Abelar J, Pharr J, Chien LC, Singh A. Estimated Car Cost as a Predictor of Driver Yielding Behaviors for Pedestrians. Journal of Transport & Health. 2020;16:100831. doi:10.1016/j.jth.2020.100831.

[4] Hennighausen C, Hudders L, Lange BP, Fink H. What If the Rival Drives a Porsche?: Luxury Car Spending as a Costly Signal in Male Intrasexual Competition. Evolutionary Psychology. 2016;14(4):1474704916678217. doi:10.1177/1474704916678217.

[5] European Commission. Council Regulation (EEC) No 4064/89 of 21 December 1989 on the control of concentrations between undertakings. Official Journal of the European Communities. 1989;32:1–12.

[6] Loureiro SMC, Kaufmann HR. Luxury values as drivers for affective commitment: The case of luxury car tribes. Cogent Business & Management. 2016;3(1):1171192. doi:10.1080/23311975.2016.1171192.

[7] Sung Y, Choi SM, Ahn H, Song YA. Dimensions of Luxury Brand Personality: Scale Development and Validation. Psychology & Marketing. 2015;32(1):121–132. doi:https://doi.org/10.1002/mar.20767.

[8] Rosecky RB, King AB. Perceptual differences among owners of luxury cars: strategic marketing implications. The Mid-Atlantic Journal of Business. 1996;32(3):221–240.

[9] Bialdyga D. Brand parity perception on the German automotive market—a comparative analysis of brand clusters. International Journal of Management Cases. 2013;15(4):265–273.

[10] Winkelmann R. Conspicuous consumption and satisfaction. Journal of Economic Psychology. 2012;33(1):183–191. doi:https://doi.org/10.1016/j.joep.2011.08.013.
